# Impact of Exercise on Gut Microbiota in Obesity

**DOI:** 10.3390/nu13113999

**Published:** 2021-11-10

**Authors:** Jerónimo Aragón-Vela, Patricio Solis-Urra, Francisco Javier Ruiz-Ojeda, Ana Isabel Álvarez-Mercado, Jorge Olivares-Arancibia, Julio Plaza-Diaz

**Affiliations:** 1 Department of Physiology, School of Pharmacy, University of Granada, Campus de Cartuja s/n, 18071 Granada, Spain; 2 Institute of Nutrition and Food Technology “José Mataix”, Center of Biomedical Research, University of Granada, Avda. del Conocimiento s/n, 18016 Granada, Spain; fjrojeda@gmail.com (F.J.R.-O.); alvarezmercado@ugr.es (A.I.Á.-M.); 3 PROFITH “PROmoting FITness and Health through Physical Activity” Research Group, Department of Physical Education and Sports, Faculty of Sports Science, University of Granada, 18071 Granada, Spain; patricio.solis.u@gmail.com; 4 Faculty of Education and Social Sciences, Universidad Andres Bello, Viña del Mar 2531015, Chile; 5 Department of Biochemistry and Molecular Biology II, School of Pharmacy, University of Granada, 18071 Granada, Spain; 6 Instituto de Investigación Biosanitaria IBS.GRANADA, Complejo Hospitalario Universitario de Granada, 18014 Granada, Spain; 7 RG Adipocytes and Metabolism, Institute for Diabetes and Obesity, Helmholtz Diabetes Center at Helmholtz Center Munich, Neuherberg, 85764 Munich, Germany; 8 Grupo AFySE, Investigación en Actividad Física y Salud Escolar, Escuela de Pedagogía en Educación Física, Facultad de Educación, Universidad de las Américas, Santiago 8370035, Chile; jorge.olivares.ar@gmail.com; 9 IRyS Research Group, School of Physical Education, Pontificia Universidad Católica de Valparaíso, Valparaíso 2374631, Chile; 10 Children’s Hospital of Eastern Ontario Research Institute, Ottawa, ON K1H 8L1, Canada

**Keywords:** gut microbiota, physical activity, health, obesity, children, humans, non-communicable diseases

## Abstract

Physical activity, exercise, or physical fitness are being studied as helpful nonpharmacological therapies to reduce signaling pathways related to inflammation. Studies describing changes in intestinal microbiota have stated that physical activity could increase the microbial variance and enhance the ratio of Firmicutes/Bacteroidetes, and both actions could neutralize the obesity progression and diminish body weight. The aim of this review is to provide an overview of the literature describing the relationship between physical activity profiles and gut microbiota and in obesity and some associated comorbidities. Promoting physical activity could support as a treatment to maintain the gut microbiota composition or to restore the balance toward an improvement of dysbiosis in obesity; however, these mechanisms need to be studied in more detail. The opportunity to control the microbiota by physical activity to improve health results and decrease obesity and related comorbidities is very attractive. Nevertheless, several incompletely answered questions need to be addressed before this strategy can be implemented.

## 1. Introduction

A positive energy balance causes excessive lipid accumulation in adipose tissue that influences the affected individual in regards to other diseases such as insulin resistance, cardiovascular diseases that can culminate in certain types of cancers and type 2 diabetes [1]. Indeed, visceral fat rather than total fat is the main driver of insulin resistance and other comorbidities such as dyslipidemia and chronic low-grade inflammation status in individuals [2]. At the cellular level, adipocyte hypertrophy results in compromised blood supply to adipose tissue, higher fibrosis, hypoxia, systemic and local inflammation, and insulin resistance in the adipocytes [3,4].

Although scientists continue making substantial progress in understanding adipose tissue function, prevention, and treatment of obesity, the pandemic continues to spread and is one of the main health complications in industrialized and developing countries [5]. In Europe, the overweight prevalence is expected to be near 50%, and the obesity prevalence to be approximately 24% of the population [6], increasing from 11% in 2000 to 24% in 2018, on typical through European member states [6,7]. It is noteworthy that high-calorie diets and the absence of physical activity, which is strongly affected by a variety of factors such as lifestyle and socioeconomic status, play a part in the advance of metabolic diseases [8].

Despite the multiple factors that interact in the progress of obesity, the gut microbiota seems to be a critical role as a bodyweight regulator [9]. In fact, Liu et al. have reported elevated body mass index (BMI) and adiposity, dyslipidemia, insulin resistance, and a more marked inflammatory status in individuals who have also exhibited significantly lesser diversity in gut bacteria [10]. Numerous mechanisms have been suggested to elucidate the potential role of gut microbiota in obesity progress.

The term microbiota includes the full collection of microbes (bacteria, fungi, and viruses, among others) that naturally subsist within a particular biological niche, around 500–1000 species, and their impact on human health [11]. Several endogenous and exogenous factors are involved in the gut microbiota such as the delivery method of a neonate, host immune response, diet, antibiotics, host genetic features, other drugs, infections, diurnal rhythm, and environmental microbial exposures [12,13].

With regards to obesity, a high-fat diet causes systemic insulin resistance and metabolic dysfunction in mice, which drives a functional and structural dysbiosis of the gut microbiota, defined as “a reduction in microbial diversity and a combination of the loss of beneficial bacteria such as *Bacteroides* strains and butyrate-producing bacteria” [12,14,15], increasing *Lactococcus* and lowering *Turicibacter*, which confer an enhanced inflammatory response [16]. The systemic inflammation in obesity is related to augmented intestinal permeability, which is linked with “leaky gut” and gut dysbiosis, which is a new objective for therapy and disease prevention [17]. Indeed, disturbances of gut microbiota composition may upsurge the leakage of the mucosa, increasing the seepage of bacteria and bacteria components such as lipopolysaccharide (LPS) across the intestinal mucosa [18]. In addition, it is well-known that obesity causes a reduction in the ratio of Bacteroidetes/Firmicutes and greater levels of *Prevotella* [19]. Thus, maintaining and restoring a healthy gut microbiota can help prevent the initial start and progress of metabolic diseases, including obesity. Microbial dysbiosis could be determined using bioinformatic analysis with ecological variables, such as diversity (alpha or beta), species richness [20], microbial abundances such as Bacteroidetes/Firmicutes ratio [21], and clinical measures such as organic acid test [22] and hydrogen breath test [23].

Numerous metabolites are created by gut microbiota from the dietary metabolism such as short-chain fatty acids (SCFAs), trimethylamine, and trimethylamine N-oxide, among others, which are linked to metabolic disorders pathogenesis [24]. In this context, new treatments (including pre and probiotics) such as fecal microbiota transplantation [25], personalized nutrition, dietary education, and physical activity [26] may help to reestablish and/or preserve the composition of gut microbiota, changing the brain–gut axis and decreasing pathology risk [27]. In particular, physical exercise induces changes in microbial diversity, cardiorespiratory fitness, and insulin resistance among others [28].

The composition of gut microbiota is directly linked to systemic metabolic homeostasis and adiposity, which influences the progress of obesity. Though several mechanisms between microbial diversity and metabolic health have been described, the causal relationship is not completely understood [29,30,31].

Exercise is recognized to stabilize the progression of obesity and modify the gut microbiota composition by increasing the microbial diversity [32,33], improving the Firmicutes/Bacteroidetes ratio that could potentially contribute to decreasing body weight, obesity-associated pathologies, and gastrointestinal disorders [32]. In addition, exercise is considered an efficient non-pharmacological therapy by decreasing inflammatory signaling pathways [34]. Overall, promoting exercise could help as a treatment to maintain the composition of gut microbiota or to restore the balance toward an improvement of dysbiosis in obesity; however, these mechanisms need to be studied in more detail. In the present work, we review the literature describing the association between exercise and gut microbiota in obesity and its associated comorbidities.

## 2. Physical Activity, Exercise, and Physical Fitness and Their Relationship with Obesity and Health Maintenance

Physical activity, exercise, and physical fitness are terms that define distinct concepts. Physical activity is well described as every physical movement using skeletal muscles that produce an energy expenditure in daily life that can be categorized into occupational, household, sports, conditioning, or other activities [35]. Exercise is a subsection of physical activity that is structured, premeditated, and cyclic and has as a final or an intermediate objective the progress or physical fitness maintenance, that is, a group of aspects that are both skills- or health-related [36].

The most in-depth and straightforward consequence associated with exercise benefits is weight loss, with the probable to mitigate or reverse the course of obesity and co-morbidities even with no dietary intervention [37,38,39]. Physical activity importance for weight loss evaluated by BMI is debated, but it is well established the effect of exercise on adiposity and several chronic diseases. Instead, there is robust evidence describing how physical activity levels and exercise intervention are important to avoid weight regain after weight loss [40,41,42,43,44,45].

According to anthropometry, there are other measures instead BMI that might be related to the gut microbiota, waist circumference, hip circumference, and waist-to-hip ratio, which have become important indicators of adiposity, disease risk, and mortality risk [46,47]. Recently, Osborne et al. have reported the associations between some of the aforementioned variables with the composition of gut microbiota. Participants with high values of BMI, mid-upper arm circumference, waist circumference, and waist-to-hip ratio were related to a diminished alpha diversity. *Oscillospira* and the family S24-7 relative abundances were inversely associated with anthropometric measures. BMI and waist and hip circumferences were related positively to *Acidaminococcus*, especially more in women than in men [47].

One of the proposed mechanisms is that physical activity raises energy expenditure activating lipolysis, so the fat mass is diminished if the energy consumed is not reimbursed for with a rise in caloric intake, maintaining a negative energy balance [48]. On the contrary, physical inactivity in contemporary obesogenic circumstances originates maladaptation, characterized by a positive energy balance, causing long-lasting disease and becoming a main public health problem [49]. Nevertheless, regular physical activity has a deep expression effect on our genome [50], which has been preferred for enhancing aerobic metabolism to maintain energy in a situation of food insufficiency [51,52], occasioning numerous favorable adaptations and reduced risk of chronic diseases [53]. The identification of adaptations of exercise is advancing our knowledge of the pathophysiology of long-lasting diseases and varying old views, which might benefit new therapeutic approaches and targets [53].

## 3. Physical Activity and Gut Microbiota in Obesity

Lifestyle changes are still the most widely used and recommended strategies to achieve weight reduction in obesity, particularly using different dietary strategies and promoting physical activity and exercise [54]. Recent authors conclude that there seems to be an intimate association between the gut microbiota and lifestyle proposing that an active lifestyle may improve the quality of the microbiota [55]. In this sense, obesity is related to minor diversity and richness of the intestinal microbiota, with a lower ratio of Bacteroidetes-to-Firmicutes [29]. Indeed, exercise training improves obesity status by increasing insulin sensitivity, reducing systemic inflammation, and improving VO_2max_ [56]. Thus, according to data from recent epidemiological, physiological, and omics-based studies, completed through studies in animals and cells studies, it seems plausible that microbial communities might be directly or indirectly influenced, among other environmental factors, by physical activity or exercise [57,58].

Maintaining or restoring microbial diversity is essential to avoid perturbed intestinal homeostasis, which is related to dysbiosis. The excessive production of SCFA, due to fermentation of non-digestible food polysaccharides by intestinal bacteria, may induce lipogenesis in the liver and accumulation of triglycerides in host adipocytes [5]. Indeed, these SCFAs may also be implicated in alterations associated with insulin resistance, given that one of the objectives for these SCFA induced signals is glucose homeostasis [59]. This is possible due to glucagon-like peptide 1 (GLP-1), a recognized mediator related to glucose homeostasis. In general terms, GLP-1 is produced in the intestine and has a critical protagonist in adjusting the plasma glucose concentration [59]. Interestingly, a recent study reveals that rectal administration of SCFAs increases the GLP-1 secretion, confirming a close association between GLP-1 and SCFAs [60]. Therefore, a fecal bacteria reduction may produce glucose intolerance through a reduction in the secretion of GLP-1 [61]. Another mechanism suggested clarifying the connection between obesity and the gut microbiota is the role of the activation of adenosine monophosphate kinase (AMPK). One of the main functions of AMPK is the oxidation of fatty acids [62]. Therefore, its inhibition could cause an increased fat accumulation [63].

## 4. Physical Activity and Microbiota: Some Underlying Mechanisms

Physical activity can stimulate variations in the gut microbiota by numerous mechanisms such as myokines release, increased intestinal transit, or the secretion of neurotransmitters and hormones [64]. However, the inter-individual variation and gut microbiota plasticity have hampered efforts to identify a “healthy” intestinal microbiota. Indeed, in addition to physical activity, many factors lead to instability of the gut microbiota, such as diet, sleep pattern, antibiotic exposure, and various comorbidities [65]. Importantly, a healthy microbiota is involved in the immune system development, with an anti-inflammatory role through reducing histone deacetylases in regulatory T cells by G protein-coupled receptors [65].

Recent studies by Castellanos et al. reported that active individuals who meet the recommendations for physical activity and diet from the World Health Organization (WHO) exhibit different relative abundances of three species of the *Bacteroides* genus: *B. uniformis*, *B. ovatus*, and an unclassified species [66]. Indeed, changes in the composition of the microbiota following an active lifestyle are associated with increases in SCFA, such as n-butyrate, which can modulate host energy balance and lead to increased nutrient availability [67]. On the other hand, a sedentary lifestyle is related to a significantly diminished diversity and a less dense microbial network structure [66]. In this context, gut microbiota diversity has developed as a candidate indicator of overall host health, the gut microbiota network of active people being more robust than that of sedentary people [64]. Thus, active people, who display a more diverse microbial community, appear to be more stable, resist pathogenic invasions, exhibit greater resilience, and show functional redundancy leading to more efficient resource utilization than sedentary individuals [68]. Furthermore, this phenomenon of increased bacterial diversity could help to restore and avoid dysbiosis, especially in severe situations such as the use of antibiotics, which this treatment per se drives a decrease of some bacterial species [66]. In concordance with these results, Bressa et al. showed that physical activity executed at short doses but continuously may rise the health-promoting bacteria abundance (*Bifidobacterium* spp., *Akkermansia muciniphila*, *Roseburia hominis*, and *Faecalibacterium prausnitzii*) in the gut microbiota [55]. However, a sedentary lifestyle is inversely associated with intestinal microbiota richness [55]. Kern et al. demonstrated that exercise increases alpha diversity and disturbs the general microbiota composition in individuals with overweight and obesity after 6 months of intervention in a randomized clinical trial (RCT) [69]. Similarly, another trial revealed that both moderate-intensity exercise and resistance training for 8 weeks trigger shifts in microbial diversity, in particular, alpha bacteria [70]. Mahdieh et al. reported an improvement of *Bifidobacterium* counts in 18 women with obesity who were training aerobic exercise (3 sessions per week, lasting 30–45 min) for 10 weeks [71]. In addition, they showed how exercise decreases intestinal inflammation and modifies gut microbiota profile in insulin-resistant subjects [72]. Therefore, physical activity is suggested as a useful non-pharmacologic instrument to counteract pathological alterations of the gut microbiota in order to prevent metabolic disturbances, especially in obesity. In addition, a sedentary lifestyle has materialized as a new risk variable for health, which is related to a high incidence of chronic diseases such as cancer cardiovascular diseases and type 2 diabetes [64,67], with obesity being the main contributor to the development of these pathologies [5].

Some proposal mechanisms of the intestinal microbiota and obesity are related to primary bile acids safely converted by gut microbiota to secondary bile acids that perform throughout the TGR5 receptor to stimulate GLP-1 release improving thermogenesis in adipose tissue [57,73] and also by the nuclear farnesoid X receptor related to carbohydrate metabolism [74].

The dietary fiber fermentation by microbes, belonging especially to Firmicutes and Bacteroidetes, creates SCFAs (propionate, butyrate, and acetate) that impact the host metabolism in several ways by performing on G protein-coupled receptors expressed by enteroendocrine cells [75,76]. SCFAs could stimulate GLP-1 and peptide YY liberation affecting the brain and pancreas, and acetate may improve fat storage through the action of ghrelin secretion [76,77].

LPS, compounds found in Gram-negative bacteria, are related to inflammation processes [77]. In addition, succinate, a microbial-derived, could have pro-inflammatory actions on LPS-derived macrophages and might therefore cause inflammation and insulin resistance in adipose tissue [78]. Other studies support that succinate could activate the uncoupling protein 1 expression that affects the adipose tissue thermogenesis [79,80]

Gut bacterial-derived branched-chain amino acids related, in cases of high fat intake, to insulin resistance in both rodents and humans [57,81]. Secreted proteins through gut bacteria also regulate endocrine or paracrine action (e.g., G protein-coupled receptor 119) [82].

Finally, physical activity could be a tentative treatment to produce different actions in specific levels, changing the microbiota composition and activating over specific proteins, such as uncoupled protein 1, or stimulating the liberation of SCFAs.

## 5. Obesity, Diet Interventions, Physical Activity/Exercise, and Their Impact on Gut Microbiota

Although scientists continue to make substantial progress in improving lifestyles in order to decrease the epidemic of obesity and its related to comorbidities, of course, lack of physical activity (strongly affected by a variety of factors, primarily socioeconomic and lifestyle status) and high-calorie diets act as an important part in the progress of metabolic diseases. The first recommended treatment for obesity is a low-calorie diet and regular physical activity. However, these lifestyle interventions have low overall compliance and limited effectiveness in people with obesity. In adults, Muralidharan et al. [31] revealed that energy restriction and Mediterranean diet with physical activity, and behavioral support could provoke a reduction in numerous Firmicutes members, especially belonging to *Lachnospiraceae* after 1-year of intervention in individuals with obesity and overweight. In addition, they observed a selective rise in some SCFA producers [31]. This type of intervention may have a beneficial effect on cardiovascular diseases, potentially modulated by gut microbiota [31]. Furthermore, Allen et al. showed that exercise-induced modulation of intestinal microbiota and SCFAs were related to changes in VO_2max_ after 6 weeks of aerobic exercise in individuals with obesity [56]. In addition, they observed that the return to the sedentary state was inversely associated with the changes that happened in response to exercise training [56]. Overall, these findings confirm that physical activity and dietary control contribute significantly to improving the composition of the gut microbiota. Moreover, it appears that exercise on fecal SCFAs levels is positively correlated with body composition [83]. One of the possible mechanisms that could explain this theory could be that SCFAs may improve skeletal muscle insulin sensitivity and regulate satiety [84], with an impact on body composition. In addition, SCFAs are also energy substrates for several tissue types, such as adipose tissue, colon, and muscle [56], suggesting that SCFAs may improve energy intake from the diet, ultimately leading to increased tissue development, including skeletal muscle [67]. Another mechanism that could explain the benefits of physical activity on the intestinal microbiota is the shift in gut pH during physical activity [83]. These changes might produce an environmental setting allowing for richer community diversity. Moreover, resistance training may cause changes in the gastrointestinal tract, as low tissue hypoxia and blood flow, driving to increased absorption capacity and transit [85].

### 5.1. Children and Adolescent Population

Interesting target population studies are children and adolescents with obesity. In this line, a recent systematic review evaluated the composition of gut microbiota related to adiposity in children and adolescents, concluding that there is an absence of consensus for the composition of intestinal microbiota associations with adiposity. Moreover, the healthy gut microbiota are related to a diet rich in fruits and vegetables with moderate consumption of animal fat and protein [86]. Of note, even the maternal gut microbiota alterations during important phases of embryonic, fetal, and early postnatal development could have consequences on the gut microbiota offspring, with lifelong effects for susceptibility to disease [87]. A recent study indicated that the diversity and abundance of gut microbiota in children with obesity were significantly minor than those in normal-weight children [88].

An exercise program combined with dietary restrictions may improve gut microbiota health in children with obesity. Nevertheless, the most effective training program is not still well established, though it seems that the combined training program of resistance and aerobic exercise could be able to decrease body fat and increase gut diversity [34,54]. Huang et al. reported that a six-week exercise training (aerobic and resistance exercises) with dietary restriction not only decreased bodyweight but also increased central hemodynamic measures (peripheral arteriolar resistance and microvascular coronary perfusion) that were related to modifying gut microbiota in adolescents with obesity [89]. In addition, a recent study has reported that six weeks of exercise and the dietary program are useful tools in order to increase autonomic function and central hemodynamics with reduced arterial stiffness in adolescents with obesity [90]. Moreover, Cho et al., with a weight reduction program that involved dietary alteration, augmented behavioral modification and physical activity during 2 months in children with obesity, observed significant modifications in the gut microbiota composition, and expected functional profiles of the gut microbiota and richness with weight loss after lifestyle changes [91]. On the other hand, Quiroga et al., without nutritional intervention, show through aerobic and resistance combined training during 12 weeks in thirty-nine children with obesity that the existence of a detrimental microbiota profile in obesity is altered by exercise intervention, emphasizing the value of exercise routine as an effective non-pharmacological therapy in early obesity [34]. Finally, in children with overweight and obesity, it is described that oligofructose-enriched inulin prebiotics modify the gut microbiota composition and moderately decrease inflammatory biomarkers, adiposity, and body weight [92].

### 5.2. Physical Activity, Diet, Microbiota, and Treatment

Physical activity significantly decreases the *Gammaproteobacteria* and *Proteobacteria.* Moreover, it tended to rise *Dialister*, *Blautia*, and *Roseburia*, creating a microbiota profile similar to that of healthy children. Training protocol significantly repressed the triggering of the obesity-associated NLRP3 signaling pathway [34]. However, there are limited findings of the gut microbiota composition in obese children, denoting that additional analysis established on the function of the intestinal microbiota in childhood obesity is necessary.

A systematic review of the outcomes of nine cross-sectional studies and six interventions reporting the outcomes of dietary fat on intestinal microbiota in humans [93] showed that diets with excessive saturated or monounsaturated fats harmfully predisposed the gut microbiota while diets high in polyunsaturated fat seemed to be neutral with respect to the gut microbiota. Similarly, high-polysaccharide diet interventions have caused different gut microbiota connected with serum, increased fecal, or urine concentrations of SCFAs, improvements of cytokine and metabolome profiles, and weight loss [94,95,96,97]. Likewise, interventions with augmented physical activity have exhibited adaptive and transmissible variations of the intestinal microbiota connected with an augmented capacity for the breakdown of branched-chain amino acids and lactate, an augmented potential for synthesis of SCFAs, and improvements in insulin sensitivity and cardiorespiratory fitness.

*Lactobacillus*, *Bifidobacterium*, and *Saccharomyces* spp. are generally recognized as safe (GRAS) probiotics [98,99,100], and in the past years, new members have been included such as *F. prausnitzii* [101], *A. muciniphila* [102], and numerous *Clostridia* spp. [103]. New data have reported that it is possible that some probiotics, *A. muciniphila* strains, do not even require colonizing the intestine to obtain helpful metabolic effects in the host related to health [104,105]. RCTs assessment of prebiotics (non-digestible polysaccharides) has reported that inulin-type fructans transformed the gut microbiota composition in adult women with obesity, leading to modest changes in host metabolism [106]. Finally, the new integrant of the “biotics” family, postbiotics, means or relates with the pasteurized version of probiotics or portions of microbial strains holding health-promoting effects [107]. A pilot trial of pasteurized *A. muciniphila* and its membrane protein Amuc_1100 showed positive effects on human metabolism indicators [102,108]. This protein recovers gut barrier functions with augmented goblet cell density by toll-like receptor 2 and moderately repeats the beneficial effect of live bacterium *A. muciniphila* on insulin sensitivity and energy metabolism [102,108].

## 6. Performance: Gut Microbiota Profile in Athletes

Professional athletes are a specific population with evident differences in microbiota composition when compared with the non-athlete population [109]. Several studies have demonstrated the association between gut microbiota and both physical activity and exercise [110]. Differences are marked also according to different athletes characteristics as well as their body composition [111]. Athlete gut microbiota shows the highest alpha diversity in comparison with obesity patients being associated with several risk factors [112]. Importantly, cardiorespiratory fitness (a powerful indicator of athletic performance and health) has been associated to Firmicutes/Bacteroidetes ratio in healthy young adults, and these associations were independent of diet composition, demonstrating that gut microbiota diversity depends on the level of physical fitness of the population [113]. On the other hand, athletes’ characteristics are also a factor related to their microbiota composition [109]. Specifically, differences are evident in rugby athletes that show a higher proportion *Akkermansia* genus than a group with an elevated BMI [33]. Moreover, the *Bacteroides* to *Prevotella* ratio is different between endurance (cardiovascular exercise performed for an extended period) training elderly subjects and healthy controls [114]. Interestingly, bacterial genus *Veillonella* was related to exercise changes in athletes after running the Boston Marathon [115], suggesting that exercise changes related to microbiota include enzymatic processes related to lactate production. Moreover, a professional cyclist shows a higher abundance in taxa related to exercise loads, such as *Prevotella* and *Methanobrevibacter smithii*, which could be related to positive consequences of exercise to performance and athletic health [45].

Finally, Donovan et al. [116] studied the microbiota composition according to different groups of sports and found differences in relative abundance across five species, namely, *Polynucleobacter necessaries*, *Eubacterium rectale*, *B. vulgatus*, *F. prausnitzii*, and *Gordonibacter massiliensis*. Interestingly, the classification was based on dynamic (more related to augmented cardiac output) and inactive components (more associated with increasing the maximal voluntary contraction) [116,117]. They found that those participating in high dynamic sport have a bigger abundance of *Lactobacillus acidophilus*, *Bifidobacterium animalis*, *F. prausnitzii*, and *Prevotella intermedia*, while athletes with both static components and high dynamic were related to a greater *Bacteroides caccae* abundance. Thus, the authors have indicated that the differences in abundance across groups may be a result of the specific characteristics of sports, principally related to the production of creatine, lactate, and substantial muscle turnover [118].

## 7. Further Perspectives

The WHO ranked physical inactivity as the fourth principal risk element for global mortality. In contrast, regular and adequate physical activity levels drop the mortality rate produced by some chronic diseases. For instance, physical exercise benefits the prevention of several obesity-related disorders such as dyslipidemia, insulin resistance, and hypertension, reducing intrahepatic lipid [119].

**Figure 1:**
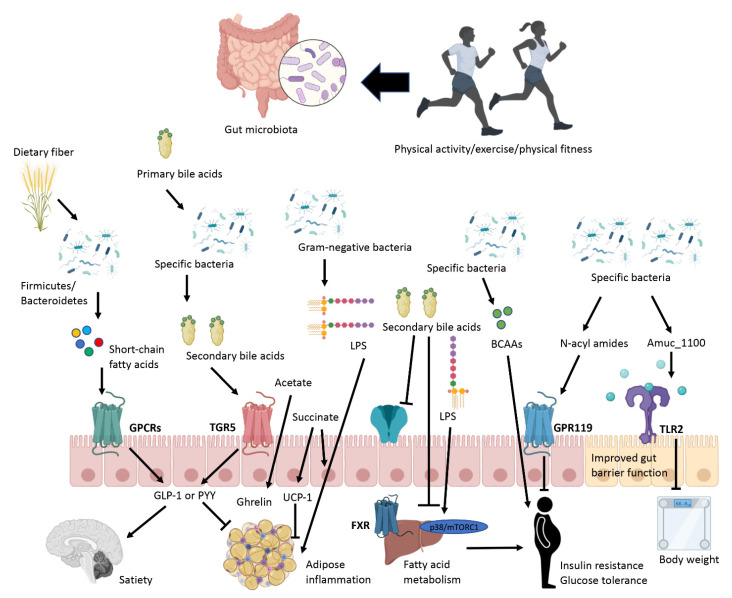
Impact of exercise on gut microbiota in obesity. Abbreviations: BCAA, branched-chain amino acids; FXR, farnesoid X receptor; LPS, lipopolysaccharide; GLP-1, Glucagon-like peptide 1; GPCRs, G-protein-coupled receptors; GPR119, G protein-coupled receptor 119; PYY, Peptide YY; UCP-1, Uncoupling protein 1; TGR5, G-protein-coupled bile acid receptor, TLR2, toll-like receptor 2.

Additionally, exercise may positively control gut microbiota in immune-based and chronic diseases. In fact, exercise can provoke qualitative and quantitative changes in microbial composition in humans [120]. In addition, a deteriorated microbiota profile may contribute to the etiopathogenesis of obesity [121]. Accordingly, there is a conceptual framework in which studies on exercise in the reversion of hypercaloric diets effects and obesity by modulating microbiota are gaining momentum within the scientific community. However, unsolved questions remain open concerning the complex nexus between microbiota, health maintenance, and obesity, and even more, the exercise intensity, type, duration, or doses remains a controversial issue. For example, it is likely the impact of exercise on the microbiota would not be the same if it is practiced as a habit to maintain weight and health, intensively as a high-level athlete, or as a weight-loss strategy with or without caloric restriction. In consequence, the exercise effects on the microbiota cannot be considered globally as there are many variables to consider in this equation depending on the ultimate purpose of the exercise. Moreover, it is also necessary to consider that exercise provokes unique microbiota profiles according to the host characteristics. Furthermore, the target exercise population should be considered, not only the baseline physical fitness and the presence or absence of underlying diseases but also the age, sex, and metabolic and hormonal status of the individual. At this point, it should be noted whether women are post- or pre-menopausal since it is one of the main controllers of circulating estrogens in the gut microbiota [122]. In consequence, the implied metabolic pathways could not be the same and therefore neither could be their impact on the microbiota. Further, the close connection between the brain and the gut microbiota (the “gut–brain axis”) should also be considered. In this regard, some pieces of evidence show that there is an elevated relationship between emotional and physical stress throughout exercise and modifications in gastrointestinal microbiota composition [123], but it is yet unsolved whether exercise modifies mental health, which is suggested by modifications in the gut–brain axis or the observed improvement in mental health due to exercise-induced changes in the microbiota. Figure 1 summarizes the main information described in the present manuscript.

## 8. Conclusions

Intestinal microbiota act as a significant player in obesity progression. Physical activity potentially benefits obesity through changes in microbiota composition. Microbiota, exercise, and dietary habits have a complex relationship. Accordingly, it is mandatory to evaluate the possible influence of exercise and specific diets, foods, nutrients, or supplements on microbial diversity in the gut. The changes of gut microbiota stimulated by physical exercise depend on the basal physiological state and maybe conditioned by parameters, such as age or BMI. Further investigation is needed to elucidate the underlying mechanisms.
